# Feasibility of Conducting Sit-to-Stand Tests Using Video Consultation

**DOI:** 10.1155/2023/8551680

**Published:** 2023-07-26

**Authors:** Deng Peng Ng, P. Thiviyan, Sailli Shrida, Li Whye Cindy Ng

**Affiliations:** ^1^Physiotherapy Department, Singapore General Hospital, Singapore; ^2^Faculty of Health & Social Sciences, School of Physiotherapy, Singapore Institute of Technology, Singapore

## Abstract

**Objective:**

This study is aimed at ascertaining the feasibility of conducting the 1-minute sit-to-stand (1MSTS) and 30-second sit-to-stand (30SSTS) tests for healthy participants via video consultation. A secondary aim was to compare the relationship between the 1MSTS and 30SSTS.

**Methods:**

A total of 63 participants were recruited via the Singapore Institute of Technology emails and social media in 2020 during the peak of COVID-19. Prior to the sit-to-stand testing, all participants completed the consent form and physical activity questionnaires. Anthropometric data such as height and weight were also collected prior to testing. An instructional video detailing the sit-to-stand (STS) movement and the requirements for the environment set-up were sent to the participants via email. All STS tests were conducted virtually via the Zoom application. Healthy participants aged 21 to 55 years old performed a 1MSTS and 30SSTS each in random order.

**Results:**

All recruited participants completed the STS tests with no reported adverse events. Majority of participants were from the 21- to 25-year-old age groups, and the average number of repetitions performed by this group was 21.9 ± 5.6 for the 30SSTS and 44.7 ± 12.6 for the 1MSTS.

**Conclusion:**

Conducting the STS tests via video consultation was demonstrated to be safe and feasible. The number of repetitions performed in the 1MSTS is correlated to that of the 30SSTS, but 1MSTS has the ability to elicit a greater HR response among younger adults.

## 1. Introduction

Exercise capacity and muscular strength and endurance are used as outcomes in predicting mortality among older adults [1, 2]. A decrease in exercise capacity indicates poor physical fitness, which predisposes individuals to chronic disease [3]. Currently, the gold standard for assessing an individual's exercise capacity is an exhaustive cardiopulmonary exercise test where the maximal oxygen consumption at peak exercise (VO_2_ max) is obtained, but expensive equipment and technical expertise are required [4]. Hence, field tests such as timed walking tests and step tests are commonly used to quantify exercise capacity and estimate muscular strength and endurance [4]. However, these field tests may not be as feasible to perform due to lack of space [5] or time [4]. The sit-to-stand (STS) test may be a more feasible alternative to assess exercise tolerance as one only needs a chair [6] and can be a safe test to conduct remotely using video consultation.

The STS action refers to going from a seated position, often from a chair, to a standing position [7]. The STS action consists of four phases—the initiation, momentum transfer, extension, and stabilisation [8]. The initiation phase involves the anterior translation of the trunk and proper positioning of the feet, and the momentum transfer is the anterior and upward translation of the centre of gravity via the dorsiflexion of the ankles [8]. The extension phase involves the extension of the hips, knees, and trunk to straighten the body, while the stabilisation phase involves the body coming upright and balanced in standing [8].

The STS action can be used to determine lower extremity performance [7] and is an important action required for activities of daily living [9]. The inability to perform a STS can severely limit an individual's mobility and quality of life [10] and is correlated to all-cause mortality [2].

### 1.1. STS Testing

The STS test is easy and quick to perform and can be done in any healthcare setting [11, 12]. Individuals are simply required to sit and stand unassisted from a secured chair, and the number of repetitions performed is recorded over a fixed period of time. The results of the test can aid a clinician in identifying individuals who are at risk of falls or have any limitation in mobility, which is a crucial component of physical function [13]. Both the 1-minute sit-to-stand (1MSTS) and 30-second sit-to-stand (30SSTS) tests challenge muscular strength and endurance [14] which are necessary for mobility [15] as well as exercise capacity.

### 1.2. The 1MSTS and 30SSTS

The 1MSTS test is a functional test that is used to measure lower limb muscular strength and endurance [7], especially in higher functioning older adults. It has a component of lactic anaerobic processes along with an aerobic component [7]. The results of the 1MSTS test are reproducible, can be used to determine exercise capacity [16], and are positively correlated to the 6-minute walk test (6MWT) [11].

The duration of the 30-second sit-to-stand (30SSTS) is half that of the 1MSTS. Therefore, the aerobic component or muscular endurance affecting an individual's performance in this test may not be as significant as the 1MSTS [6]. The repetitions performed in the 30SSTS have been found to be an effective measure of physical performance due to its correlation with lower limb muscle power [17]. However, normative data for 30SSTS are only available among older adults above the age of 60 [18–20].

### 1.3. Video Consultation

In the climate of the unprecedented COVID-19 pandemic, many countries around the world went into lockdowns, and people were advised to stay at home to curb the spread of the virus. Under these circumstances, healthcare workers were required to find alternative methods of assessing and treating patients in need of healthcare services from their homes. One such method was telemedicine. The results of a review by Tenforde et al. [21] demonstrated the feasibility and effectiveness of telemedicine in the management of certain neurological and musculoskeletal conditions.

This study is aimed at establishing the feasibility of conducting 1MSTS and 30SSTS in healthy participants and reviewing the relationship between 1MSTS and 30SSTS.

## 2. Methodology

### 2.1. Study Design

This is a cross-sectional study conducted among 63 adults between ages 21 and 55, across six months.

### 2.2. Recruitment

Healthy participants aged 21-55 who were able to perform the STS test independently were recruited through emails, social media, and advertisements, such as posters. Selected participants were asked to provide an email address, following which they were sent a digital consent form and information sheet, along with a Physical Activity Readiness Questionnaire (PAR-Q) [22] and the short form of the self-reported International Physical Activity Questionnaire (IPAQ) [23]. In addition to this, a link to an instructional video was included in the email. The video contained instructions on the set-up for the STS tests to ensure the safety of the participants (the appendix). Finally, a pamphlet consisting of three lower limb stretches—hamstrings, quadriceps, and calf stretches—was also included in the email.

The IPAQ provides a brief overview of the activity level of an individual within a week. Individuals are required to fill in the duration of their vigorous, moderate, and walking activities within the last seven days, as well as the number of hours spent sitting on a weekday. All these activities are used to calculate the multiples of metabolic equivalent (MET) minutes per week, which indicate the physical activity level of a participant in a week. A score of 600 MET minutes per week indicates that an individual is minimally active, while 3000 MET minutes per week indicates a health-enhancing physically active (HEPA) level, which is the desired level of physical activity per week [23].

Participants with musculoskeletal issues that prevent them from carrying out as many STS independently, prior surgery over the last 3 months, or having existing cardiac conditions were excluded from the study. Once participants were deemed fit to perform the tests, they were advised to reply to the email, and a scheduled date and time was made to perform the test virtually via Zoom. Participants were also encouraged to clarify any doubts or ask any questions regarding the tests via email.

The assessors met each participant virtually via Zoom on the agreed date and time. During the Zoom call, participants were asked to provide their smoking history, race, height, and weight prior to testing. The information was recorded in a data collection sheet. Participants were informed of the order of the tests they would be performing and were allowed to clarify any doubts before the tests. The Zoom calls were not recorded.

The 1MSTS and 30SSTS tests were performed based on standardised instructions (see the appendix), with a 15-minute break between the tests. A randomised generator, https://www.randomizer.org, was used to randomise the order of the tests to eliminate any bias that may arise because of the test order. Participants only performed each test once as there is no learning effect for the STS test [24, 25]. Each participant reported their heart rate, Borg's rate of perceived exertion (RPE) score, and dyspnoea score before and after each test.

Heart rate was measured using either a heart rate monitor or via palpation of the radial pulse over the wrist by the participants. A stopwatch was used to time both the 1MSTS and 30SSTS. Participants were encouraged to use a chair that was 46 cm high with no armrests. The chair was placed against a wall for safety purposes. Participants were asked to place their camera in front of the test area and far enough to allow the assessors to view the entire STS sequence.

The participant sat with the knees and hips flexed to 90°, feet placed flat on the floor hip-width apart, and the hands placed across the chest. All participants were given the same standardised instructions (the appendix). The STS repetition was considered invalid should participants use their hands to assist in completing any repetitions. The number of proper STS repetitions was recorded by the assessor during the live Zoom session using a hand-held counter. The STS repetition that was deemed improper was not considered in the final repetition score. Participants were encouraged to perform the lower limb stretches from a pamphlet that was sent earlier to them to prevent muscle soreness.

### 2.3. Data Analysis

The Statistical Package for the Social Sciences version 26 was used for all statistical analysis. Multiple linear regression was used to compare various variables with the number of STS repetitions performed with no adjustments for potential confounders. Independent *T* tests were also performed to compare the change in heart rate with the number of repetitions of STS per second.

### 2.4. Ethics

The study was approved by the Singapore Institute of Technology ethics board (number 2020036). Anthropometric data collected was encrypted and stored.

## 3. Results

### 3.1. Participants

The 63 participants included in the study had a median age of 24 years. The sample contained 57% male participants, and majority of the participants were between the 21- and 25-year-old age groups.  Table 1 shows the demographic and anthropometric data of the participants. The MET minutes per week in Table 1 were calculated using the IPAQ scoring guidelines, and participants who indicated “not sure” in any of the sections were excluded from the calculation of physical activity.

There were no significant correlations found between age and repetitions for either test (*r* = −0.114 for the 1MSTS, *r* = 0.113 for the 30SSTS). There was insignificant correlation found between physical activity and number of repetitions performed (*r* = 0.069 for the 1MSTS, *r* = −0.076 for the 30SSTS). Finally, there was also no correlation found between BMI and repetitions performed (*r* = −0.058 for the 1MSTS, *r* = 0.114 for the 30SSTS). There was a statistically significant difference between repetitions performed between the gender, with male participants performing significantly more repetitions than female participants in both the 1MSTS and the 30SSTS (*p* < 0.05).

**Figure 1:**
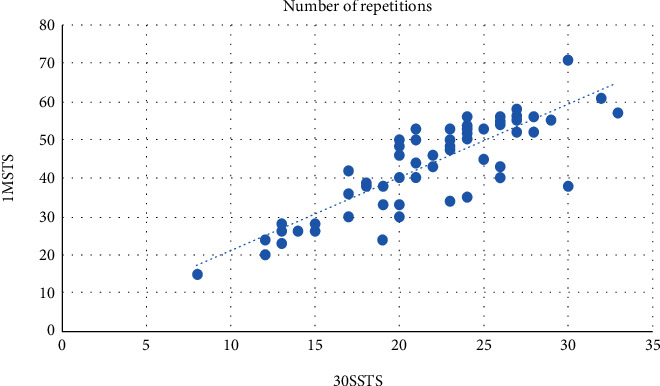
Relationship between 30SSTS and 1MSTS. A linear correlation between the number of repetitions performed for both tests is observed (*r* = 0.84). 1MSTS: 1-minute sit to stand; 30SSTS: 30-second sit to stand.

In Table 2, 3.2% of participants reported an RPE score of 4 and above in the 30SSTS, while 11.1% of participants reported an RPE score of 4 and above in the 1MSTS. Tables 3 and 4 show the number of STS repetitions based on age bands and gender, respectively. The largest number of participants was between 21 and 25 years old.  Figure 1 shows a linear relationship between the number of repetitions performed during the 30SSTS and 1MSTS.  Table 5 shows that the 1MSTS elicited a significant heart rate response compared to the 30SSTS.

## 4. Discussion

The STS tests can be conducted and assessed safely with clear instructions provided via video consultation. The 30SSTS is strongly correlated with 1MSTS in younger healthy adults. Furthermore, the 1MSTS appears to elicit greater heart rate responses, indicating a possible alternative for assessing exercise capacity remotely via video consultation.

Most of the participants in the study were considered HEPA, as they were meeting the criteria of a minimum of 3000 MET minutes per week. This implied that they were exceeding the minimum physical activity recommendation for a healthy lifestyle [23]. Most of the participants in the study rated both the 30SSTS and 1MSTS to be below an RPE score of 6, which indicate a perception of low to moderate intensity level. This indicates that majority of the participants felt that the 30SSTS and 1MSTS tests were of low-moderate exercise intensity, with a higher percentage of participants rating the 1MSTS between 4 and 6. This is further observed as there was no statistically significant difference in the rate of repetitions of STS regardless of timing. As a moderate intensity score of 4 to 6 on the RPE correlates to 40-59% VO_2_ max of an individual [26], the 1MSTS can possibly be used as a submaximal fitness test. This finding correlates to the systematic review that states that the 1MSTS can be used to quantify submaximal exercise capacity in a home health setting [16], likely from the greater metabolic demand and oxygen consumption.

As a higher BMI does not correlate to a better physical performance among college-aged students [27], the results of this study are consistent in showing that there is no significant correlation between BMI of participants and repetitions performed in the 30SSTS and 1MSTS. No significant correlation was seen between physical activity levels and repetitions performed by participants. This may have been due to the younger and physically active participants recruited.

Male participants in the study did significantly more repetitions than female participants in both the 30SSTS and 1MSTS. This finding was consistent with the results of other studies [18, 20, 28]. This may be due to females having a lower fat-free mass than males [29]. A lower fat-free mass is associated with a poorer exercise capacity [30]. Hence, the fewer repetitions performed by female participants in the 1MSTS can be attributed to their lower fat-free mass and a smaller size of muscle fibres, both of which correlate to reduced muscle strength [31]. Women are also shown to perform more poorly than men in submaximal cardiovascular exercise [32]. As the 1MSTS is a submaximal test, this may also explain the fewer repetitions performed by female participants in the 1MSTS.

In comparison with data from a Caucasian sample [28], participants from this study had significantly fewer repetitions for age-matched participants in the age group between 21 and 25. The mean repetitions performed by the participants in this study were lower than the 50^th^ percentile of the age-matched participants in the Swiss study [28]. This difference in performance may have been due to the differences in body composition between Asian and Caucasian individuals [33]. A study by Poh et al. [34] investigated the differences between the 6-minute walk test performances between Singaporean Chinese and Dutch Caucasian populations. The Dutch Caucasian participants had a better performance, and this was attributed to their higher fat-free mass and increased height and leg length [34]. This study thus concluded that the six-minute walk distance in healthy Singaporean adults cannot be predicted using reference equations derived from Caucasian populations. As the results of the 6MWT are correlated to the 1MSTS [14], the contributing factors leading to a poorer performance in the 1MSTS by Asian participants may be similar.

In the review by Bohannon and Crouch [16], future research suggested included investigating the suitability of the 1MSTS for submaximal exercise capacity testing in a home health setting. This was especially important as other commonly used tests, such as the 6MWT, are not as feasible to perform due to logistical requirements. The 1MSTS has been found to be a good tool to evaluate exercise capacity even in patients with heart failure [35] and pulmonary diseases [36]. No incidents or adverse events were reported during the Zoom sessions in this study, suggesting that it is safe to conduct the STS remotely under virtual supervision. There are recent studies where STS assessments were conducted remotely, but 30SSTS [37, 38] or 5 times STS [39] were chosen. As 1MSTS can be used to quantify submaximal exercise capacity, this study has demonstrated that it can be conducted safely in a remote manner.

The strength of this study is that there were no dropouts or adverse events; all participants recruited completed both tests. Further, this study was able to demonstrate feasibility in conducting a STS assessment in a home environment using video consultation, with the ability to elicit appropriate cardiovascular responses from each participant. This was all made possible by having clear instructions and ensuring safety measures were taken in the home environment.

### 4.1. Limitations

The recruitment of participants was done online and via university channels. This might explain the younger and more tech-savvy participants recruited. In addition, the test was conducted in the participants' home, making it difficult to standardise the heart rate monitor and chair used. As participants were required to self-declare their height, weight, and physical activity, this data may be subjected to bias.

## 5. Conclusion

It is safe and easy to carry out sit-to-stand tests via video consultation, and the findings can be used to determine lower limb muscle endurance. The number of repetitions from the 1MSTS is correlated to that of the 30SSTS, but 1MSTS was able to elicit a greater HR response among younger adults. Both sit-to-stand tests are safe to be conducted remotely under video consultation where safety measures include clear instructions given by a video and having the chair leaned against the wall.

## Figures and Tables

**Table 1:** Demographic data of the participants (*n* = 63).

Mean age (years)	26.1 ± 7.3	
Gender (M/F)	36/27	
Average BMI (kg/m^2^)	21.5 ± 2.6	
Smoking habits	Smokers	8
	Nonsmokers	55
Ethnicity (%)	Chinese	40 (63.5)
	Malay	4 (6.3)
	Indian	16 (25.4)
	Others	3 (4.8)
MET minutes/week^∗^	3791.8 ± 2492.1	
Seated hours/day^^^	7.9 ± 3.2	

^∗^Data of 20 participants who declared “unsure” was excluded. ^^^Data of 31 participants who declared “unsure” was excluded. M: male; F: female; BMI: body mass index; MET: metabolic equivalents.

**Table 2:** RPE intensities reported after each test by percentage of participants.

		RPE		
		Low (0-3)	Moderate (4-6)	High (7-10)
30SSTS (*n* = 63)	Number of participants (%)	61 (96.8)	2 (3.2)	0
1MSTS (*n* = 63)	Number of participants (%)	56 (88.9)	6 (9.5)	1 (1.6)

RPE: rate of perceived exertion; 30SSTS: 30-second sit to stand; 1MSTS: 1-minute sit to stand.

**Table 3:** Number of repetitions obtained for the 30SSTS and 1MSTS tests based on age band.

Participant age	Number of participants	30SSTS mean repetitions	1MSTS mean repetitions
21-25	48	21.9 ± 5.6	44.7 ± 12.6
26-30	8	21.4 ± 2.3	42.9 ± 8.2
31-35	2	21 ± 2.8	40 ± 10.0
36-40	0	0	0
41-45	1	24	53
46-50	1	15	26
51-55	3	25 ± 5.0	38 ± 5

30SSTS: 30-second sit to stand; 1MSTS: 1-minute sit to stand.

**Table 4:** Mean repetitions performed in each test by different gender.

Test	Gender	Repetitions performed (mean ± SD)
30SSTS	Male	23.2 ± 4.6
	Female	20.1 ± 5.4
1MSTS	Male	48.3 ± 9.7
	Female	38.0 ± 12.1

30SSTS: 30-second sit to stand; 1MSTS: 1-minute sit to stand.

**Table 5:** Heart rate responses.

	Repetitions (second)	Baseline HR	Change in HR	Percent of HRR
30SSTS	0.73 ± 0.17	78.76 ± 10.56	20.70 ± 15.32	18.03 ± 12.80
1MSTS	0.73 ± 0.20	79.78 ± 8.16	31.49 ± 14.17	27.80 ± 12.00
*p* value	0.92	0.28	<0.05	<0.05

1MSTS: 1-minute sit to stand; 30SSTS: 30-second sit to stand; HR: heart rate; HRR: heart rate reserve.

## Data Availability

The data collected is stored under Singapore Institute of Technology one-drive and is word-protected. Anthropometric data collected was encrypted and stored.

## Appendix

### A. Standardised Instructions

#### A.1. Instructions for Video Consultation

Please ensure that you have a firm chair, with a seat height of approximately 46 cm. Ensure the chair has no armrests.

Lean the chair against the wall during the test.

Please place the camera in front of the test area and far enough to allow the assessors to view the entire STS sequence

#### A.2. Standardised Instructions

The purpose of the test is to assess your exercise capacity and leg muscle strength. Please sit on the chair with the knees and hips flexed to 90° and feet placed flat on the floor hip-width apart, with your hands placed across the chest. The movement required is to get up from this chair with the legs straight and sit back continuing the repetitions as fast as possible within one minute. The assisted use of the arms is not allowed during the test. I will give you the countdown “3, 2, 1 Go” as an indication to start, and I will also tell you when we are at the 15 remaining seconds. If required, you can make a break or rest and resume the test as soon as possible, as the goal is to complete as many sit-to-stand cycles as possible in 30 seconds/one minute (^∗∗^to select the right period time).

When I say “3, 2, 1 Go,” please begin.
